# Personalizing AI tools for second language speaking: the role of gender and autistic traits

**DOI:** 10.3389/fpsyt.2024.1464575

**Published:** 2025-01-27

**Authors:** Yiran Du, Chenghao Wang, Bin Zou, Yinan Xia

**Affiliations:** ^1^ Institute of Cognitive Neuroscience, University College London, London, United Kingdom; ^2^ Department of Applied Linguistics, Xi’an Jiaotong-Liverpool University, Suzhou, China

**Keywords:** gender difference, autistic traits, artificial intelligence (AI), second language (L2), speaking

## Abstract

**Introduction:**

It is important to consider individual differences in research on educational technology. This study investigates the interplay between autistic traits, gender, and the perception of artificial intelligence (AI) tools designed for second language (L2) speaking practice, contributing to a deeper understanding of inclusive educational technology.

**Methods:**

A sample of 111 university students completed the Broad Autism Phenotype Questionnaire (BAPQ) to measure autistic traits (AU) and their sub-traits Aloof (AF), Rigid (RD), and Pragmatic Language (PL). Perceptions of AI tools were assessed across five dimensions: Perceived Usefulness (PU), Perceived Ease of Use (PEOU), Attitude (AT), Behavioral Intention (BI), and Usage Behavior (UB). The study utilized correlation and regression analyses to examine relationships between these variables, while exploring gender-specific moderating effects.

**Results:**

Key findings revealed no significant gender differences in autistic traits or overall perceptions of AI tools. Contrary to expectations, autistic traits were negatively correlated with perceptions of AI tools, suggesting that current AI designs may not adequately support individuals with pronounced autistic traits. Additionally, gender moderated some relationships, with males displaying stronger associations between autistic traits and both PEOU and UB.

**Discussion:**

This research bridges critical gaps by linking neurodiversity and gender to technology acceptance, advancing the field’s understanding of individual differences in AI-based language learning. It underscores the importance of designing personalized and adaptive educational tools that address diverse learner needs, promoting inclusivity and effectiveness in L2 practice.

## 1 Introduction

Autism spectrum disorder (ASD) is increasingly recognized not only as a clinical diagnosis (1–3) but also in terms of sub-threshold traits that are present to varying degrees in the general population (4). These autistic traits can influence various aspects of life, including academic performance and social interactions. Recent research has begun to explore the nuances of these traits, including their distribution across genders and their impact on educational performances in higher education settings (e.g., 5, 6). Parallel to the exploration of autistic traits, the advent of artificial intelligence (AI) tools in education, particularly in language learning, presents a new frontier for enhancing the learning experience of second language (L2) learners. AI-driven tools, leveraging technologies such as voice recognition and automatic speech recognition (ASR), offer personalized and accessible opportunities for language practice, potentially benefiting a wide range of learners, including those with autistic traits. However, the intersection of autistic traits, gender differences, and the utilization and perception of AI tools in education has not been thoroughly examined. This study seeks to bridge these gaps by examining the gender differences in autistic traits among neurotypical university students and how these traits may influence the perceptions and effectiveness of AI tools for L2 speaking practice. By investigating these relationships, the research aims to contribute to a more nuanced understanding of how individual differences affect language learning in technological environments and to foster more inclusive and effective educational strategies. This exploration is particularly timely, given the increasing prevalence of ASD and the rapid advancement and adoption of AI in education.

### 1.1 Autistic traits and gender

ASD is identified as a neurodevelopmental condition marked by challenges in social interaction and communication, alongside repetitive behaviors and interests (7) and the prevalence of ASD is increasing significantly (1–3). A significant characteristic of ASD is the gender disparity in its prevalence, with a higher occurrence observed in males compared to females (8–10). Despite the known male predominance in autism diagnoses, research on the link between gender differences and ASD, covering clinical, neurobiological, and etiological aspects, is multifaceted and often yields complex, unrelated findings, leaving several critical questions about their relationship unexplored (11, 12). Core behavioral features of ASD are known to differ widely in intensity and range, with a growing acknowledgment that numerous individuals exhibit traits akin to ASD symptoms which are called autistic traits, even if they don’t meet the formal diagnostic threshold (4). Initially observed in the parents and close relatives of children with autism, these ‘sub-threshold’ autistic traits have since been recognized as widely spread throughout the general population (13–15), hinting at the idea that autism might be viewed as a spectrum of traits present to varying degrees in people at large (4). These traits can hinder the academic success of students who exhibit complete or partial signs of ASD in higher education settings (5). For example, students exhibiting more pronounced autistic characteristics which covers areas like social and communication skills, attention shifting, and imagination deficits—have reported experiencing higher levels of loneliness and lower quality social relationships compared to their university peers (6). Moreover, Lu et al. (5), involving 1381 Chinese college students, highlights the significant role of emotional regulation and friendships in mediating the negative impact of autistic traits on school adaptation, suggesting the need to focus on these areas to improve college experiences for students with such traits. The relasionship between autistic traits and academic performance can be influenced by gender. For example, Hsiao et al. (16) explored the impact of autistic-like social deficits on school and social functioning among 1321 students from Grades 1 to 8 in northern Taiwan, highlighting the moderating role of gender. Findings indicated that these deficits are linked to various adjustment issues, with boys showing a stronger association between autistic-like traits and negative school attitudes, social problems at school, and difficult peer relationships compared to girls.

In summary, previous research has explored the relationship between autistic traits and academic success, including the role of gender, suggesting potential educational implications. However, investigations in this domain remain scarce. Therefore, this study aims to explore how autistic traits influence Computer-Assisted Language Learning (CALL) among neurotypical university students, focusing on their perceptions of AI tools for L2 speaking practice.

### 1.2 AI tools for L2 speaking practice

In recent times, AI has been integrated in CALL (17) and AI tools has shown educational and psychological benefits for practicing L2 speaking (18). For example, one study explored the impact of AI-powered speech evaluation systems on the speaking skills of English as a Foreign Language (EFL) learners (19). Forty participants from China engaged in the research, which involved both qualitative and quantitative data collection. The findings indicate that most learners felt their speaking abilities improved due to the feedback from the AI evaluation program. Additionally, there was a notable increase in their speaking skill scores between pre- and post-tests. The study suggests enhancing AI evaluation systems with diverse textual feedback and actionable advice to better support EFL learners in improving their speaking skills. Moreover, Bashori et al. (20) examined Foreign Language Speaking Anxiety (FLSA) among Indonesian vocational high school students and assessed whether web-based language learning, particularly with ASR technology, could mitigate this anxiety. Involving 573 students with a focused experiment on 167 participants using ASR-based platforms, the research found that these students experienced moderate to severe FLSA. However, the use of web-based language learning platforms was viewed positively by the students, who believed these tools could reduce their speaking anxiety. Interviews with students and teachers suggested that speaking to ASR-based websites was less intimidating than speaking in front of peers or people, indicating potential benefits of ASR technology in alleviating FLSA and making language learning more accessible and less stressful. Given the benefits, many AI tools for speaking practice has been increasingly popular (21), such as Duolingo (22), Liulishuo (23), EAP Talk (24), ChatGPT (25), Call Annie (26), etc.

Despite the body of research in CALL which includes AI tools for speaking practice, the focus on individual differences has been somewhat limited, with researchers concentrating more on areas such as beliefs, learning strategies, self-regulation, motivation, and willingness to communicate (27). In contrast, other factors such as gender have received less attention, and the impact of autistic traits in the context of CALL and AI tools for speaking practice remains entirely unexplored.

### 1.3 The present study

In summary, the literature reveals significant gaps in the study of autistic traits, gender differences, and the application of AI tools for L2 speaking practice. There is a notable lack of research on the nuanced differences in autistic traits between male and female neurotypical university students and how these traits might impact academic performance. Furthermore, while the educational benefits of AI tools in L2 speaking practice are acknowledged, there is a deficiency in thorough investigations into how individual differences, such as gender and autistic traits, affect the efficacy and perception of these technologies. The convergence of autistic traits, gender disparities, and the use of AI tools for language learning highlights a critical research void, emphasizing the need for comprehensive studies to develop more personalized and inclusive educational approaches. To bridge these gaps, this study will explore the gender differences in autistic traits, perceptions of AI tools for L2 speaking practice, and their interrelations among neurotypical male and female university students. Specifically, the study aims to address the following research questions:

1. How do male and female students differ in their perceptions of AI tools for L2 speaking practice?
2. What differences exist in autistic traits between male and female students?
3. How do autistic traits relate to perceptions of AI tools for L2 speaking practice?
4. Does gender influence the relationship between autistic traits and the perceptions?

### 1.4 Predictions

Given the higher rates of ASD diagnosis among males (8–10) and the higher scores of autistic traits among males in the general population (13), it is anticipated that neurotypical male university students will exhibit higher levels of autistic traits. Despite these differences in autistic traits, based on literature suggesting no significant differences in perceptions, attitudes, and uses of computers between genders (e.g., 28), this study hypothesizes that there will be no significant differences in perceptions of AI tools for L2 speaking practice between male and female students. Moreover, this study predicts that autistic traits will positively relate to students’ perceptions of AI tools for L2 speaking practice. This is based on the notion that individuals with higher autistic traits may prefer structured and rule-based interactions, similar to those with ASD (4). However, due to a lack of prior research in this specific area, it is challenging to predict the impact of gender on the relationship between autistic traits and perceptions of AI tools for L2 speaking practice.

## 2 Methodology

This study adopted an open science approach (29). It was pre-registered in Open Science Framework: https://osf.io/qtpxf. The data, data analysis code, and research materials are available at: https://osf.io/hycqf.

### 2.1 Participants

174 participants were recruited using convenience and snowball sampling methods (30). 64 of them were excluded because they either never used AI tools for speaking practice before or detected as careless respondents in survey using minimal level screening methods suggested by Ward and Meade (31), resulting in 111 valid responses. The number of participants exceeded the minimal sample size determined through power analysis using G*Power (32). Specifically, for the multiple linear regression, a sample size of 103 participants is required to detect a moderate effect size (*f²* = 0.15), with an alpha level of 0.05 and a power of 0.80, considering 7 predictors (see Section 3.3). Demographic data (see **Table 1**) revealed a predominance of female participants, constituting 72.1% (*n* = 80), while males comprised 27.9% (*n* = 31). The majority of participants fell within the 18-22 age range, accounting for 94.6% (*n* = 105). Education levels varied, with most being freshmen (69.4%, *n* = 77). In terms of academic major, participants were almost evenly divided between STEM (51.4%, *n* = 57) and non-STEM fields (48.6%, *n* = 54). Usage of AI tools was led by ChatGPT with 83.8% (*n* = 93) of participants using it, followed by other tools like EAP Talk (30.6%, *n* = 34) and Shanby (28.8%, *n* = 32). English was the most common L2 among participants, practiced by 89.2% (*n* = 99), and the frequency of AI tool use was most commonly “seldom” (43.2%, *n* = 48) or “sometimes” (39.6%, *n* = 44).

**Table 1:** Demographic information of participants.

Variables	Items	Frequency	Percentage
Gender	Male	31	27.9%
	Female	80	72.1%
Age	Below 18	3	2.7%
	18-22	105	94.6%
	23-26	3	2.7%
Education level	Freshman	77	69.4%
	Sophomore	17	15.3%
	Junior	15	13.5%
	Senior	1	0.9%
	Postgraduate	1	0.9%
Major	STEM	57	51.4%
	Non-STEM	54	18.6%
AI tools	ChatGPT	93	83.8%
	Call Annie	20	18.0%
	EAP Talk	34	30.6%
	Liulishuo	13	11.7%
	Duolingo	25	22.5%
	Shanby	32	28.8%
	Babbel	3	2.7%
L2	English	99	89.2%
	Spanish	61	55.0%
	Japanese	28	25.2%
	Korean	3	2.7%
	French	1	0.9%
	German	2	1.8%
Frequency of use	Seldom	48	43.2%
	Sometimes	44	39.6%
	Often	13	11.7%
	Always	6	5.4%

### 2.2 Measures

#### 2.2.1 The broad autism phenotype questionnaire

The Broad Autism Phenotype Questionnaire (BAPQ; 33) was utilized to assess autistic traits (AU). Initially designed to detect traits resembling autism in relatives of autistic individuals, the BAPQ has been extended to assess these traits in both autistic and non-autistic populations as well (e.g., 34, 35). The BAPQ calculates scores for the entire survey and three distinct sub-scales: Aloof (AF), Pragmatic Language (PL), and Rigid (RD). The Aloof sub-scale measures the extent of social withdrawal and a reserved demeanor, Pragmatic Language evaluate challenges in social communicative skills, and Rigid focuses on the lack of flexibility in actions and thinking processes. Responses to each BAPQ item are scored on a 6-point scale, from ‘‘very rarely’’ to ‘‘very often’’. The questionnaire generates an overall score along with scores for the three subdomains by averaging the responses across the total 36 items and the 12 items within each subdomain, resulting in summary scores that span from 1 to 6. The whole scale (Cronbach’s α = 0.84) and the Aloof scale (Cronbach’s α = 0.88) had good reliability (36). However, Cronbach’s α values for the Rigid and Pragmatic Language were 0.66 and 0.64 respectively.

#### 2.2.2 Perceptions

The study examined participants’ perceptions across five dimensions. Specifically, it assessed perceived usefulness (PU), which refers to the degree to which participants believe that utilizing AI tools enhances their speaking skills, using a modified scale from Zou et al. (19). It also evaluated perceived ease of use (PEOU), the degree to which participants feel that using AI tools requires minimal effort, and their attitude (AT) which refers to the extent to which learners are interested in and positively assess the use of AI tools for their speaking practice, both of which were adapted from scales by Liu and Ma (37). Furthermore, the research looked into behavioral intention (BI), or the likelihood that participants would independently use AI tools for practicing speech, with modifications from a scale by Abbad (38). The study also incorporated a Usage Behavior (UB) scale, adapted from Abbad (38). This scale specifically measured the extent of learners’ independent engagement with AI tools for speaking practice. The scale adaption was based on suggestions from Du (39). Each of these scales was based on a 5-point Likert scale, ranging from 1 (strongly disagree) to 5 (strongly agree), and demonstrated good reliability with Cronbach’s α values between 0.75 and 0.85 (36).

#### 2.2.3 Procedure

Participants were asked to finish a questionnaire via a Qualtrics-hosted URL link (https://www.qualtrics.com). They were briefed about the voluntary nature of their involvement, the option to withdraw at any time, and the confidentiality and research-only use of their data before starting. A consent form, requiring their agreement to proceed, was presented initially. The questionnaire initiated with demographic queries, followed by instructions to complete the BAPQ. Subsequently, their perceptions on using AI tools for L2 speaking practice were examined. Upon finishing, participants received a debriefing and appreciation for their participation.

### 2.3 Data analysis

In this study, quantitative analysis was conducted using R (Version 4.3.2). Descriptive statistics were calculated for the questionnaire data. Welch’s independent samples t-tests were then conducted to explore differences in autistic traits and perceptions of using AI tools for practicing L2 speaking between neurotypical university males and females. This test is well-suited to handle situations with unequal group sizes and variances (40). Correlation tests were conducted to explore the relationships between autistic traits and the perceptions. Finally, to investigate how gender influences these relationships, multiple linear regression models were employed with a dummy variable, Male (0 = female, 1 = male). The models: PU/PEOU/AT/BI/UB ~ AU + Male + AU: Male/AF + RD + PL + Male + AF: Male + RD: Male + PL: Male were constructed. The reason for not including AU and its subscales AF, RD, and PL in one model is to avoid multicollinearity. The coefficients (*b*_1_) of AU, AF, RD, and PL indicate their effects on the dependent variables (i.e., PU, PEOU, AT, BI, UB) for females (since Male = 0). Specifically, they show how much the dependent variables are expected to change with a one-unit change in AU, AF, RD, or PL for females. The coefficients of the interaction terms (*b*_2_) (i.e., AU: Male, AF: Male, RD: Male, PL: Male) indicate whether the effects differ between males and females and how these effects change when the individual is male. Specifically, for males, *b*_1_ + *b*_2_ units change in the dependent variables are associated with one unit change in AU, AF, RD, or PL. The statistical significance of their effects on the dependent variables for males was examined by F-tests using the linearHypothesis function from the car package. The regression coefficients (*b*), their standard errors (*SE*), 95% CI, t-values, and significance levels for each model were presented (see **Table 1**).

## 3 Results

### 3.1 Gender differences in autistic traits and perceptions of using AI tools

In examining gender differences among neurotypical university students regarding autistic traits and perceptions of using AI tools for L2 speaking practice, Welch’s independent samples t-tests yielded the following findings (see **Table 2**): For autistic traits (AU), no significant differences were found between males (*M* = 3.09, *SD* = 0.43) and females (*M* = 3.07, *SD* = 0.49), *t*(61.49) = 0.20, *p* = 0.842. Similarly, there were no significant differences in Aloof (AF) (Males: *M* = 3.25, *SD* = 0.84; Females: *M* = 3.22, *SD* = 0.80; *t*(52.53) = 0.18, *p* = 0.857) and Rigid (RD) (Males: *M* = 3.20, *SD* = 0.52; Females: *M* = 3.17, *SD* = 0.54; *t*(57.34) = 0.25, *p* = 0.805). Pragmatic Language (PL) were also comparable (Males: *M* = 2.83, *SD* = 0.48; Females: *M* = 2.84, *SD* = 0.56; *t*(63.87) = -0.02, *p* = 0.984).

**Table 2:** Results of Welch’s independent samples t-tests.

	Gender	*M*	*SD*	95% CI		*df*	*t*
				2.5%	97.5%		
AU	Male	3.09	0.43	-0.17	0.21	61.49	0.20
	Female	3.07	0.49				
AF	Male	3.25	0.84	-0.32	0.38	52.53	0.18
	Female	3.22	0.80				
RD	Male	3.20	0.52	-0.19	0.25	57.34	0.25
	Female	3.17	0.54				
PL	Male	2.83	0.48	-0.21	0.21	63.87	-0.02
	Female	2.84	0.56				
PU	Male	3.86	0.53	-0.04	0.40	49.97	1.61
	Female	3.69	0.47				
PEOU	Male	3.91	0.70	-0.03	0.56	53.62	1.80^+^
	Female	3.65	0.69				
AT	Male	3.82	0.59	-0.18	0.33	59.57	0.59
	Female	3.75	0.65				
BI	Male	3.88	0.59	-0.28	0.29	60.15	0.01
	Female	3.88	0.65				
UB	Male	3.23	0.94	-0.23	0.55	49.25	0.84
	Female	3.07	0.84				

^+^ *p* < 0.1.

Regarding perceptions of AI tools, the Perceived Usefulness (PU) indicated a non-significant trend towards higher scores for males (*M* = 3.86, *SD* = 0.53) compared to females (*M* = 3.69, *SD* = 0.47), *t*(49.97) = 1.61, *p* = 0.113. Perceived Ease of Use (PEOU) was marginally higher for males (*M* = 3.91, *SD* = 0.70) than for females (*M* = 3.65, *SD* = 0.69), *t*(53.62) = 1.80, *p* = 0.078. No significant differences emerged in Attitudes (AT) towards using AI tools (Males: *M* = 3.82, *SD* = 0.59; Females: *M* = 3.75, *SD* = 0.65; *t*(59.57) = 0.59, *p* = 0.557), Behavioral Intentions (BI) (Males: *M* = 3.88, *SD* = 0.59; Females: *M* = 3.88, *SD* = 0.65; *t*(60.15) = 0.01, *p* = 0.994), or Usage Behavior (UB) (Males: *M* = 3.23, *SD* = 0.94; Females: *M* = 3.07, *SD* = 0.84; *t*(49.25) = 0.84, *p* = 0.407).

### 3.2 Relationships between autistic traits and perceptions of AI tools

The results of the correlation analysis between traits associated with autism and perceptions of AI tools for L2 speaking practice are insightful (see **Table 3**). Autistic Traits (AU) displayed negative correlations with PU at *r* = -0.21, *p* < 0.05, PEOU at *r* = -0.18, *p* < 0.1, and AT at *r* = -0.25, *p* < 0.01. There was a nonsignificant correlation between AU and both BI and UB. Aloof (AF) showed similar negative trends, with correlations of *r* = -0.16, *p* < 0.1 with PU, and nonsignificant with PEOU, AT, BI, and UB. Rigid (RD) was negatively correlated with PU at *r* = -0.17, *p* < 0.1, PEOU at *r* = -0.21, *p* < 0.05, AT at *r* = -0.32, *p* < 0.01, and BI at *r* = -0.23, *p* < 0.05, but showed no significant relationship with UB. Lastly, Pragmatic Language (PL) displayed negligible correlations with perceptions. These findings highlighted how specific autistic traits correlate differently with perceptions towards AI tools for L2 speaking practice, potentially influencing their acceptance and use.

**Table 3:** Results of correlation tests.

	AU	AF	RD	PL	PU	PEOU	AT	BI	UB
AU	1.00	0.84***	0.65***	0.73***	-0.21*	-0.18^+^	-0.25 **	-0.15	0.01
AF		1.00	0.30**	0.43***	-0.16^+^	-0.10	-0.16^+^	-0.10	-0.00
RD			1.00	0.28**	-0.17^+^	-0.21*	-0.32***	-0.23*	-0.12
PL				1.00	-0.14	-0.12	-0.10	-0.01	0.15
PU					1.00	0.41***	0.58 ***	0.45 ***	0.31 **
PEOU						1.00	0.54 ***	0.34 ***	0.32 ***
AT							1.00	0.72 ***	0.57 ***
BI								1.00	0.58 ***
UB									1.00

^+^ *p* < 0.1, *p < 0.05, **p < 0.01, ***p < 0.001.

### 3.3 The influence of gender on the relationships between autistic traits and perceptions of AI tools

Several multiple linear regression models were conducted to explore how the relationship between autistic traits and perceptions of AI tools for L2 speaking practice differed between males and females (see **Table 4**). In the first model for Perceived Usefulness (PU), the coefficient for Autistic Traits (AU) was 0.30 with a p-value < 0.01, indicating a statistically significant negative effect on PU. This suggests that for females, each one-unit increase in autistic traits was associated with a decrease of 0.30 in PU. However, the interaction term AU: Male was not statistically significant, indicating that gender had no effect on the relationship between PU and AU. In the second PU model, the coefficient for Aloof (AF) was -0.13 with a p-value approaching significance (*p* < 0.1). This near-significant negative coefficient suggests that AF may decrease PU, particularly in females, with each unit increase leading to a decrease of 0.13 in PU. However, the interaction term AF: Male did not show significant effects, indicating that gender does not significantly alter the influence of AF on PU.

**Table 4:** Results of multiple linear regression models.

	Predictors	*b*	*SE*	95% CI		*t*
				2.5%	97.5%	
PU	Intercept	4.61	0.34	3.93	5.29	13.42***
	AU	-0.30	0.11	-0.52	-0.08	-2.72**
	Male	-0.88	0.72	-2.31	0.55	-1.22
	AU: Male	0.34	0.23	-0.12	0.80	1.48
PU	Intercept	4.56	0.38	3.81	5.31	12.1***
	AF	-0.13	0.08	-0.29	0.03	-1.67^+^
	RD	-0.09	0.11	-0.31	0.12	-0.86
	PL	-0.05	0.12	-0.28	0.18	-0.45
	Male	-0.56	0.81	-2.17	1.05	-0.69
	AF: Male	0.23	0.14	-0.05	0.50	1.65
	Rigid: Male	-0.10	0.21	-0.51	0.32	-0.47
	PL: Male	0.11	0.22	-0.33	0.56	0.50
PEOU	Intercept	4.55	0.49	3.58	5.52	9.32***
	AU	-0.29	0.16	-0.61	0.02	-1.88^+^
	Male	-0.07	1.03	-2.10	1.96	-0.07
	AU: Male	0.11	0.33	-0.54	0.76	0.33
PEOU	Intercept	4.54	0.52	3.51	5.57	8.73***
	AF	-0.10	0.11	-0.32	0.12	-0.92
	RD	-0.09	0.15	-0.38	0.21	-0.59
	PL	-0.10	0.16	-0.42	0.21	-0.63
	Male	0.82	1.12	-1.40	3.04	0.74
	AF: Male	0.30	0.19	-0.08	0.68	1.58*
	Rigid: Male	-0.62	0.29	-1.20	-0.05	-2.15
	PL: Male	0.16	0.31	-0.45	0.78	0.53
AT	Intercept	5.25	0.43	4.40	6.10	12.22***
	AU	-0.49	0.14	-0.76	-0.21	-3.54^+^
	Male	-2.06	0.90	-3.84	-0.27	-2.28
	AU: Male	0.69	0.29	0.12	1.27	2.40
AT	Intercept	5.46	0.46	4.54	6.37	11.84***
	AF	-0.15	0.10	-0.35	0.05	-1.52
	RD	-0.40	0.13	-0.66	-0.14	-3.02*
	PL	0.01	0.14	-0.27	0.29	0.09
	Male	-2.09	0.99	-4.06	-0.12	-2.10
	AF: Male	0.28	0.17	-0.06	0.61	1.66
	Rigid: Male	0.18	0.26	-0.33	0.69	0.71
	PL: Male	0.24	0.27	-0.30	0.79	0.89
BI	Intercept	4.69	0.45	3.80	5.59	10.35***
	AU	-0.27	0.15	-0.55	0.02	-1.82^+^
	Male	-0.91	0.95	-2.80	0.97	-0.96
	AU: Male	0.30	0.30	-0.31	0.90	0.98
BI	Intercept	4.89	0.49	3.92	5.86	9.97***
	AF	-0.08	0.10	-0.28	0.13	-0.73
	RD	-0.31	0.14	-0.59	-0.03	-2.19*
	PL	0.07	0.15	-0.22	0.37	0.49
	Male	-1.14	1.06	-3.23	0.96	-1.08
	AF: Male	0.09	0.18	-0.26	0.45	0.51
	Rigid: Male	0.11	0.27	-0.43	0.66	0.42
	PL: Male	0.17	0.29	-0.41	0.75	0.58
UB	Intercept	3.50	0.62	2.28	4.73	5.68***
	AU	-0.14	0.20	-0.53	0.25	-0.71
	Male	-2.03	1.29	-4.60	0.54	-1.57
	AU: Male	0.71	0.42	-0.11	1.53	1.71^+^
UB	Intercept	3.66	0.65	2.37	4.95	5.62***
	AF	-0.07	0.14	-0.35	0.21	-0.5
	RD	-0.27	0.19	-0.64	0.10	-1.44
	PL	0.17	0.2	-0.22	0.57	0.87
	Male	-2.75	1.4	-5.53	0.03	-1.96^+^
	AF: Male	0.14	0.24	-0.33	0.61	0.59
	Rigid: Male	0.00	0.36	-0.72	0.72	0.01
	PL: Male	0.87	0.39	0.10	1.63	2.24*

^+^ *p* < 0.1, *p < 0.05, **p < 0.01, ***p < 0.001.

In the first model for Perceived Ease of Use (PEOU), AU was negatively associated with PEOU for females (b = -0.29), and this relationship was marginally statistically significant (*p* < 0.10). Specifically, a one-unit change in AU was associated with a -0.29 unit change in PEOU. However, the relationship between PEOU and AU was not moderated by gender, as indicated by the non-significant effect of the AU: Male interaction.

The second PEOU model showed that the interaction term AF: Male had a significant positive effect on PEOU (*b* = 0.30, p < 0.05). This indicates that gender moderated the relationship between PEOU and AF, being more pronounced in males. For males, a one-unit change in AF was associated with a 0.20 unit change in PEOU (*b*_AF: Male_ + *b*_AF_ = 0.30 – 0.10 = 0.20). However, this positive correlation for males was not statistically significant (*F*(1, 103) = 1.63, *p* = 0.20).

In the first model for Attitude (AT), AU was negatively associated with AT for females (*b* = -0.49), and this relationship was marginally statistically significant (*p* < 0.10). Specifically, a one-unit change in AU was associated with a -0.49 unit change in AT. However, the relationship between AT and AU was not moderated by gender, as indicated by the non-significant effect of the AU: Male interaction. The second AT model showed that Rigidity (RD) was negatively correlated with AT for females, with the relationship being statistically significant (*p* < 0.05). Specifically, a one-unit change in RD was associated with a -0.40 unit change in AT. Again, the relationship was not affected by gender, as indicated by the non-significant effect of the RD: Male interaction.

In the first model for Behavioral Intention (BI), AU was negatively associated with BI for females (*b* = -0.27), and this relationship was marginally statistically significant (*p* < 0.10). Specifically, a one-unit change in AU was associated with a -0.27 unit change in BI. The relationship between BI and AU was not moderated by gender, as indicated by the non-significant effect of the AU: Male interaction. The second BI model showed that Rigidity (RD) was negatively correlated with BI in females (*p* < 0.05). Specifically, a one-unit change in RD was associated with a -0.31 unit change in BI. Again, the relationship was not affected by gender, as indicated by the non-significant effect of the RD: Male interaction.

The first model for Usage Behavior (UB) indicated that the interaction term AU: Male was positively associated with UB (*b* = 0.71), with this relationship being marginally statistically significant (*p* < 0.10). This suggested that gender moderated the relationship between UB and AU, being more pronounced in males. Specifically, for males, a one-unit change in AU was associated with a 0.57 unit increase in UB (*b*_AU: Male_ + *b*_AU_ = 0.71 – 0.14 = 0.57). However, this positive correlation was not statistically significant for males (*F*(1, 107) = 2.44, *p* = 0.12). The second UB model showed that the interaction term PL: Male was also positively associated with UB (b = 0.71), and this relationship was marginally statistically significant (p < 0.10). This indicates that gender impacted the relationship between Pragmatic Language (PL) and UB, again more pronounced in males. Specifically, for males, a one-unit change in PL was associated with a significant increase of 0.57 units in UB (*b*_PL: Male_ + *b*_PL_ = 0.71 – 0.14 = 0.57), with this effect being statistically significant (*F*(1, 103) = 9.77, *p* < 0.01).

## 4 Discussion

The study explored differences in Autistic Traits (AU) and sub-traits—specifically, Aloof (AF), Rigid (RD), and Pragmatic Language (PL) — and perceptions of AI tools for L2 speaking practice between neurotypical males and females. It also examined the relationships between the traits and perceptions of these tools, including Perceived Usefulness (PU), Perceived Ease of Use (PEOU), Attitude (AT), Behavioral Intention (BI), and Usage Behavior (UB). Additionally, the study investigated whether gender moderated these relationships.

Results showed no significant differences of AU, AF, RD, and PL between males and females. They were against the prediction that neurotypical male university students will exhibit higher levels of autistic traits due to the higher rates of ASD diagnosis among males (8–10) and the higher scores of autistic traits among males in the general population (13). However, Ruzich et al. (13) used the Autism-Spectrum Quotient (AQ) (41) to measure autistic traits and the current study utilied the Broad Autism Phenotype Questionnaire (BAPQ; 33), which might cause the different results. The difference may also be due to the current study’s specific focus on university students rather than the general population.

Results also showed no significant differences in PU, AT, BI, and UB between males and females, although males demonstrated marginally higher scores in PEOU than females. These findings are aligned with previous research that showed no significant differences in perceptions, attitudes, and uses of computers between genders (28). However, more recent research indicated that males hold more positive attitudes toward the use of technology compared to females, albeit with small effect sizes (42).

Moreover, this study found that AU was negatively correlated with PU, AT, and PEOU (although only marginally). AF was also marginally negatively correlated with PU and AT. Additionally, RD was negatively correlated with PEOU, AT, and BI, and marginally negatively correlated with PU. These findings contradict the predictions that autistic traits would be positively correlated with students’ perceptions of AI tools for L2 speaking practice. This prediction was based on the assumption that individuals with higher autistic traits might prefer structured and rule-based interactions, similar to those with ASD (4). The reasons for these findings may be that current AI tools are more interactive and humanized, thanks to advancements in generative AI (43).

The current study also found evidence of gender’s moderating role in the relationship between autistic traits and perceptions. Specifically, the relationships between PEOU and AF, UB and AU, and UB and PL were more pronounced in males. This may be due to the complex effects of various factors such as cognitive and neurological differences, differing socialization and learning styles, variability in autistic traits presentation between genders, differential impacts of autistic sub-traits, societal gender roles and expectations, and differential sensitivity to technology features.

However, several potential limitations can be identified, which may influence the interpretation and generalizability of the findings. One of the primary concerns is the method of participant recruitment, which utilized convenience and snowball sampling methods. These approaches are prone to selection bias as they may not adequately represent the entire student population, potentially limiting the applicability of the conclusions to broader, more diverse groups of students. The demographic skew (72% female, 94.6% aged 18–22) limits the representativeness of the findings. Another significant limitation arises from the measurement tools used, particularly the Broad Autism Phenotype Questionnaire (BAPQ). While the BAPQ is a validated instrument, the lower Cronbach’s alpha values for its RD and PL sub-scales suggest issues with internal consistency, which could affect the reliability of findings related to these specific traits. This measurement challenge underscores the need for future research to either refine existing instruments or develop new tools that ensure higher reliability across different dimensions of autistic traits. Additionally, the cross-sectional design of the study restricts the ability to draw causal inferences from the data. The relationships identified between autistic traits, gender, and perceptions of AI tools could be better understood through longitudinal research that tracks changes over time, or experimental designs that can directly test the influence of specific variables. Moreover, the study does not address how cultural or educational contexts might influence perceptions of AI tools or the presentation of autistic traits. Lastly, the rapid pace of technological advancement in AI and educational tools poses a challenge to the long-term relevance of the study’s findings. As AI technologies evolve, so too may their acceptance and perceived utility among students, which suggests the need for ongoing research to monitor these changes.

In conclusion, this study explored the interplay of gender differences, autistic traits, and the use of AI tools for L2 speaking practice among neurotypical university students, contributing valuable insights into the nuanced impacts of these variables on educational technology utilization. Contrary to expectations, the results revealed no significant differences in autistic traits between genders or in the overall perceptions of AI tools for L2 speaking practice. However, subtle variations were observed in certain perception measures, like PEOU, where males scored marginally higher than females. The findings suggest that autistic traits negatively correlate with certain perceptions of AI tools, contradicting the initial hypothesis that higher autistic traits would lead to more favorable perceptions due to a presumed preference for structured and rule-based learning environments. This could indicate a potential mismatch between the design of current AI tools and the needs of individuals with pronounced autistic traits, emphasizing the necessity for more personalized educational technologies. Furthermore, gender was found to moderate some relationships between autistic traits and perceptions of AI tools, particularly in UB and PEOU, suggesting that male students may experience these aspects differently compared to female students. This underscores the complexity of individual differences in educational technology engagement and highlights the importance of considering these factors in the design and implementation of AI tools. The study’s implications extend beyond the academic setting, influencing future educational strategies, AI tool development, and policy-making to ensure inclusive, effective, and personalized learning environments for all students, regardless of gender or neurodiverse traits. By addressing the specific needs and preferences of individuals, educational technologies can enhance learning outcomes and better support diverse student populations. This research lays a groundwork for further studies into the intricate dynamics of autism traits, gender differences, and technology use in education, advocating for a continuous refinement of educational tools to meet the evolving demands of learners.

## Data Availability

The datasets presented in this study can be found in online repositories. The names of the repository/repositories and accession number(s) can be found below: https://osf.io/hycqf.
